# Virucidal Effect of the Mesoscopic Structure of CAC-717 on Severe Acute Respiratory Syndrome Coronavirus-2

**DOI:** 10.3390/microorganisms9102096

**Published:** 2021-10-04

**Authors:** Takashi Yokoyama, Tomoyasu Nishimura, Yoshifumi Uwamino, Kenjiro Kosaki, Koichi Furusaki, Rumiko Onishi, Takashi Onodera, Makoto Haritani, Katsuaki Sugiura, Rikio Kirisawa, Naoki Hasegawa

**Affiliations:** 1 Environmental Science for Sustainable Development, Graduate School of Agricultural and Life Sciences, The University of Tokyo, Tokyo 113-8657, Japan; atonode@g.ecc.u-tokyo.ac.jp (T.O.); aharitani@g.ecc.u-tokyo.ac.jp (M.H.); aksugiur@g.ecc.u-tokyo.ac.jp (K.S.); 2 Department of Infectious Diseases, Keio University School of Medicine, Tokyo 160-8582, Japan; tnishimura@keio.jp (T.N.); uwamino@keio.jp (Y.U.); n-hasegawa@z8.keio.jp (N.H.); 3 Center for Medical Genetics, Keio University School of Medicine, Tokyo 160-8582, Japan; kkosaki@keio.jp; 4 Mineral Activation Technical Research Center, Kumamoto 865-0023, Japan; furusaki@ind.bbiq.jp; 5 Santa Mineral Co., Ltd., Tokyo 105-0013, Japan; rumiko@santa-mineral.co.jp; 6 School of Veterinary Nursing and Technology, Faculty of Veterinary Science, Nippon Veterinary and Life Science University, Tokyo 180-8602, Japan; 7 Nippon Institute for Biological Science, Tokyo 198-0024, Japan; 8 Laboratory of Veterinary Virology, Department of Pathobiology, School of Veterinary Medicine, Rakuno Gakuen University, Hokkaido 069-8501, Japan; r-kirisa@rakuno.ac.jp

**Keywords:** SARS-CoV-2, disinfectant, mesoscopic structure, CAC-717

## Abstract

Here, the virucidal effect of calcium bicarbonate with a mesoscopic structure (CAC-717) on severe acute respiratory syndrome coronavirus-2 (SARS-CoV-2) was determined. Assays showed that CAC-717 had a strong virucidal effect on all examined SARS-CoV-2 isolates, including variant strains. The viral infectivity decreased within 15 s, and the virucidal activity of CAC-717 at a 1:49 ratio was similar to that of ethanol disinfectant. CAC-717 neutralization eliminated this virucidal effect, indicating that the alkaline condition of CAC-717 is important for virus inactivation and is an indicator of its mesoscopic structure and virucidal activity. The virucidal effect was observed in the presence of organic matter (bovine serum albumin). CAC-717 is a non-invasive and non-flammable substance with a low environmental burden, and its usefulness as a novel disinfectant has been confirmed.

## 1. Introduction

Coronavirus disease 2019 (COVID-19), caused by severe acute respiratory syndrome coronavirus 2 (SARS-CoV-2), threatens public health and society worldwide. Public health measures that ensure personal protection, such as social distancing, wearing masks, maintaining hand hygiene, and testing for the early diagnosis of infection, have been implemented [1]. Reducing the number of viruses in the environment is integral to controlling infection; therefore, effective cleaning and disinfection, particularly in medical institutions and nursing homes, is critical. Numerous disinfectants are effective against SARS-CoV-2 [2], and ethanol and sodium hypochlorite exhibit remarkable antiviral activity. However, ethanol is flammable and requires adequate ventilation, and sodium hypochlorite is inactivated in the presence of organic substances, high temperature, and sunlight and might generate chlorine gas via reactions with other chemicals. Sodium hypochlorite is also a strong skin and mucous membrane irritant. Disinfectants must be used at an optimal concentration and exposure time to ensure effectiveness; however, excessive spraying and use present health and environmental hazards [3]. As efforts to inactivate SARS-CoV-2 are undertaken in a variety of places and circumstances, non-invasive disinfectants exhibiting low toxicity and low environmental burden are desired.

A macroscopically uniform material appears complex on the mesoscopic scale, which is intermediate between the microscopic and macroscopic sample dimensions [4], with corresponding pore sizes of 2–50 nm [5]. The performance of mesoscopic materials is determined by crystal and/or domain networks, such as topology, correlation length, ordering, and linkage strength [6,7]. Mesoscopic structures create new collective properties and can be utilized as artificial atoms for building new materials [7]. Calcium carbonate is one of the most intensively investigated chemicals because of its widespread use [8]. CAC-717 (Santa Minerals Co. Ltd., FDA/USA Regulation No. 880.6890, Class 1 disinfectant, Japan Patent No. 5778328) contains 6.9 mM calcium bicarbonate and exhibits a mesoscopic structure and novel properties. CAC-717 is a strong alkaline solution that exhibits antimicrobial effects on noroviruses [9] and influenza viruses [10] and inactivates prions [11]. In this study, we aimed to verify the virucidal effect of CAC-717 against SARS-CoV-2 and confirm its usefulness as a novel disinfectant.

## 2. Methods

### 2.1. Cell Lines and Virus Strains

VeroE6/TMPRSS2 cells expressing the transmembrane serine protease 2 gene and showing high susceptibility against SARS-CoV-2 [12] were obtained from the Japanese Collection of Research Bioresources Cell Bank and cultured in Dulbecco’s modified Eagle’s medium (DMEM, Nacalai, Kyoto, Japan) supplemented with 5% fetal bovine serum (FBS) and penicillin streptomycin. SARS-CoV-2/WK-521 [12], hCoV-19/Japan/QK002/2020 (alpha variant: EPI_ISL_804008), hCoV-19/Japan/TY7-501/2021 (beta variant: EPI_ISL_833366), and hCoV-19/Japan/TY8-612/2021 (gamma variant: EPI_ISL_1123289) were obtained from the National Institute of Infectious Diseases, Japan. Furthermore, isolated viruses (SARS-CoV-2/KH-1 and SARS-CoV-2/KH-25/2021 [delta variant]) from throat swabs of patients with COVID-19 at Keio Hospital were also used as trend strains. Isolated viruses were identified as SARS-CoV-2 via real-time polymerase chain reaction with the 2019 Novel Coronavirus Detection kit (Shimadzu Co., Ltd., Kyoto, Japan), and mutations were validated by whole genome sequencing (submitted). The virus was propagated in VeroE6/TMPRSS2 cells, maintained in DMEM supplemented with 5% FBS, and stored at −80 °C until use. All experiments were performed in a biosafety Level 3 laboratory with permission from the Biosafety Committee of Keio University School of Medicine.

### 2.2. Median Tissue Culture Infectious Dose (TCID_50_) Assay

Confluent VeroE6/TMPRSS2 cells, which were seeded 1 day before, were incubated for 1 h at 37 °C in 96-well plates with 100 μL of 10-fold serially diluted sample (see the following sections). Thereafter, 100 µL of DMEM supplemented with 5% FBS was added to each well. The cells were incubated at 37 °C for 7 d. The cytopathic effect was monitored and the median TCID_50_ was determined.

### 2.3. Virus Inactivation with CAC-717

CAC-717 (pH 12.39 ± 0.03) [10] was provided by Santa Mineral Co., Ltd. (Tokyo, Japan). For analysis, 12 μL of virus sample was mixed with 108 μL (1:9 dilution), 588 μL (1:49 dilution) or 1188 μL (1:99 dilution) of CAC-717 and subsequently incubated for 15, 30, 60, or 300 s at 20 °C. Thereafter, the samples were mixed with 120 μL of 1 M 4-(2-hydroxyethyl)-1-piperazineethanesulfonic acid buffer (HEPES, pH 7.6) to terminate the CAC-717 effect, and 120 μL of 7.5% NaCl solution was added to adjust the osmotic pressure. The samples (1:9 and 1:49 dilution) were subsequently diluted to 1200 μL with distilled water. The total volume of 1:99 dilution was 1440 μL. Subsequently, 100 μL of each sample was used to inoculate the cells. Following 1 h of incubation with the sample, 100 µL of DMEM supplemented with 5% FBS was added. For the mock control, 12 μL of the virus was mixed with 588 μL of distilled water and incubated for 300 s at 20 °C. The mixture was then mixed with 120 μL of 1 M HEPES and 120 μL of 7.5% NaCl solution and diluted to 1200 μL. Thereafter, the samples were serially diluted and used as inocula. In the case of a titer lower than the detection limit of the assay, possible maximum titers were fitted. The difference in the titers of samples with/without CAC-717 was calculated and indicated as the viral reduction rate.

### 2.4. Virus Inactivation with Commercial Disinfectant

A commercial ethanol disinfectant (ethanol solution for disinfecting Hakuzo EI, Hakuzo Medical Asia Co. Ltd., Osaka, Japan), which was composed of 83% ethanol and 3.7% isopropanol, was used as a control. A mixture consisting of 108 μL of ethanol solution and 12 μL of virus sample was incubated for 15, 30, 60, or 300 s at 20 °C. Thereafter, 120 μL of 1 M HEPES and 120 μL of 7.5% NaCl solution were added and the volume was made up to 1200 μL with distilled water.

### 2.5. Effect of pH on Virus Activity

DMEM of various pH levels (5–13) was prepared with 1 M HCl or 10 N and 1 N NaOH. Following pH adjustment, DMEM was passed through a 0.2µm filter (Millex-LG., Millipore). Twelve microliters of SARS-CoV-2 virus stock (WK-521) with a concentration of 10^7.3^ TCID_50_/mL was mixed with 108 μL of pH-adjusted DMEM (1:9 dilution) and incubated at room temperature for 5 min. Thereafter, 1080 µL of DMEM (pH 7.5) without FBS was mixed, and 1:9 serial dilutions with DMEM were prepared and plating was performed.

### 2.6. Virucidal Effect of CAC-717 with Organic Material

As an organic matter, 5% bovine serum albumin (BSA, Bovine Serum F-V, Nacalai) was used. It was centrifuged (Kubota, 3520, Tokyo, Japan) at 14,000× *g* for 10 min and subsequently passed through a 0.45μm filter before use. Twelve microliters of SARS-CoV-2 virus stock (WK-521) at a concentration of 10^7.3^ TCID_50_/mL was mixed with 108 μL of BSA and incubated for 5 min at 20 °C. Thereafter, these samples were mixed with 1080 µL of DMEM or CAC-717 (1:9 dilution) and incubated for 5 min at 20 °C. CAC-717 was neutralized with 120 µL of HEPES.

### 2.7. Statistical Analysis

Paired *t*-tests were employed to compare the virucidal effect between CA-717 and commercial ethanol using Microsoft Excel. Differences were considered significant *p* < 0.05.

## 3. Results

### 3.1. Time and Dose Effects of CAC-717

As a control, 10^5.0^ TCID_50_/mL of the virus was detected in the samples used in this experiment. We observed that the 9-, 49- and 99-fold doses of CAC-717 showed a distinct virucidal effect. The virucidal effect of the 99- and 49-fold doses were marginally higher than that of the 9-fold dose. The virucidal effect of the 9-fold dose was associated with time-dependent sensitization. However, the 99- and 49-fold doses did not show any difference in terms of sensitization time. A distinct inactivation effect was observed with the use of commercial ethanol solution. The virucidal effect of ethanol was marginally higher at a 1:9 ratio (significant difference was observed at 30 s); however, the virucidal effect of CAC-717 at the 99- and 49-fold doses was similar to that of the commercial ethanol solution (Table 1).

### 3.2. HEPES Inhibits the Virucidal Effect of CAC-717

As CAC-717 shows strong alkalinity, HEPES was used to terminate the reaction. To confirm its effect, CAC-717 was mixed with HEPES before addition to the virus solution. Here, 10^4.8^ TCID_50_/mL virus was detected after treating CAC-717 with HEPES. The pH of HEPES-treated CAC-717 was 7.6 and its virucidal effect disappeared. Thus, HEPES neutralization effectively terminated the CAC-717 reaction (Table 1).

### 3.3. Virucidal Efficacy of CAC-717 on Different Strains

There are various mutant strains of SARS-CoV-2. In addition to the original virus (WK-521), virucidal effects of CAC-717 on the alpha, beta, gamma, and delta variants were investigated. A virucidal effect against all strains was confirmed, and the log_10_ reduction in infectivity was ≥3.6 to ≥4.4 (Table 2).

### 3.4. Effect of pH on SARS-CoV-2 Viability

Given that the inactivation effect of CAC-717 was due to high alkalinity, the viral titers in DMEM of different pH levels were analyzed. Similar viral titers were observed at pH 5 to 10. The infectious titer was attenuated at pH 11 and 12. However, viral infectivity was typically reduced at pH 12.4, which is similar to the pH of CAC-717 (Table 3).

### 3.5. Effect of Organic Materials on CAC-717 Activity

The effect of the presence of organic matter on CAC-717 was analyzed to verify the virucidal effect in the general environment. The infectious titers of the virus mixed with 5% BSA was 10^4.8^ TCID_50_/mL. The inactivation effect of CAC-717 was observed and its log reduction was ≥ 4.3 (Table 4).

## 4. Discussion

CAC-717 rapidly reduced the infectivity of SARS-CoV-2, as reported previously [13]. Here, we report the detailed results and additional characteristics of CAC-717. The virucidal effect against recent variant strains was also confirmed. The virucidal effect of CAC-717 was similar to that of ethanol. The strong alkalinity of CAC-717 is considered important for virus inactivation. This hypothesis is supported by the observation that HEPES-neutralized CAC-717 loses its virucidal effect. SARS-CoV-2 reportedly loses its infectivity at pH 13 [14]. We also confirmed that the virus survives in a wide range of pH values (pH 5–12); however, the virus was clearly inactivated at pH 12.4 and likewise with CAC-717.

HEPES, which has a strong buffering ability, can neutralize CAC-717 such that it loses its virucidal activity. This indicates that CAC-717 can be used as long as alkaline conditions persist. Under strong acidic conditions, prewashing with water and/or the use of several volumes of CAC-717 might be recommended.

To confirm the suitability of CAC-717 for sanitizing dirty areas such as toilets, we evaluated its activity in the presence of organic materials. It has known that hypochlorite is rapidly inactivated in the presence of organic materials. However, the virucidal effect of CAC-717 was not diminished in the presence of organic materials.

It has also been reported that CAC-717 exhibits antimicrobial effects on norovirus [9] and influenza viruses [10] and inactivates prions [11]. Here, we showed its virucidal effect on all examined SARS-CoV-2 isolates, including recent mutant strains.

Research indicates that mesoscopic structures create novel properties via electromagnetic interactions. CAC-717 might exhibit dielectric properties and electrolyze the surrounding water molecules. Its strong alkalinity could be due to the relative decrease in H^+^, owing to its accumulation within the structure, which leads to a relative increase in OH^−^. The high alkalinity of CAC-717 might be caused by increased OH^−^, and the absence of other ions, such as Na^+^, is thought to result in the low toxicity of CAC-717. Alkaline conditions are thought to be an indicator of the mesoscopic structure and virucidal activity of CAC-717 and could be used for quality control of products. In addition, a small titer of virus was detected for 30 s at pH 12.4 (Table 3). Thus, it is possible that the unidentified property of the mesoscopic structure might also be responsible for the virucidal effect. Further studies are necessary to clarify the characteristics of mesoscopic structures.

No safe chemicals exist, and consequently, there is a need to develop disinfectants that are less harmful to humans and the environment [15]. Large amounts of disinfectants have been used for COVID-19 control; thus, attention should be paid to future environmental damage. CAC-717 has been shown to be harmless and does not cause skin toxicity and eye toxicity in rabbits [10]. Given that the calcium bicarbonate component of CAC-717, which is derived from plant minerals, is non-flammable, it could be used in a variety of applications for which ethanol is not suitable. Recently, the virucidal effect of commercial ophthalmic solutions was indicated and propose the potential use of preventing ocular infection [16]. CAC-717 is not approved as a medical drug, however, it is less irritating, and may also be a potential disinfecting agent. Further research and validations are necessary in future.

This study revealed that the mesoscopic structure CAC-717 has a strong virucidal effect on all examined SARS-CoV-2 isolates within 15 s. The virucidal activity of CAC-717 was similar to that of a commercial ethanol disinfectant, and HEPES neutralization eliminated this effect. The experiment was conducted in a BSL3 laboratory, which is a fully controlled environment with defined conditions. The stability of CAC-717 on different types of surfaces should thus be evaluated. In this study, we demonstrated the usefulness of CAC-717 under laboratory conditions. It is necessary to verify the disinfecting effect of CAC-717 in the medical environment and/or social circumstances.

## Figures and Tables

**Table 1:** Time- and dose-dependent effects of CAC-717 on SARS-CoV-2 viability.

Reagent	Ratio ^1^	Virus Titers (TCID_50_/mL)			
		15 s	30 s	60 s	300 s
CAC-717	1:9	≤1.0 ± 0.4 ^2^	≤0.8 ± 0.2 *	≤0.7 ± 0.2	≤0.6 ± 0.1
	1:49	≤0.6 ± 0.1	≤0.7 ± 0.1	≤0.6 ± 0.1	≤0.5 ± 0.1
	1:99	≤0.7± 0.1	≤0.7 ± 0.1	≤0.6 ± 0	≤0.6 ± 0
Control	1:49	ND	ND	ND	5.0 ± 0.1
HEPES-treated CAC-717 ^3^	1:49	ND	ND	ND	4.8 ± 0.2
Alcohol disinfectant	1:9	≤0.5 ± 0	≤0.5 ± 0.1	≤0.5 ± 0	≤0.5 ± 0

The titer of virus (SARS-CoV-2/KH-1/2021) used in this experiment was 10^6.8 ± 0.3^ TCID_50_/mL. ^1^ Twelve microliters of virus solution was mixed with 9, 49 or 99 volumes of reagent. For control, 49 volumes of distilled water was used. ^2^ Data are mean ± standard deviation of four independent experiments. The maximum possible viral titers are shown. ^3^ HEPES neutralized the alkalinity of CAC-717, and the pH was changed from 12.4 to 7.6. * A significant difference as compared with alcohol disinfectant (*p* < 0.05).

**Table 2:** Virucidal efficacy of CAC-717 against SARS-CoV-2 variants.

Strain	Variant Type	Viral Titers (TCID_50_/mL)		Log_10_ Reduction ^1^
		Distilled Water	CAC-717 Treatment (1:49)	
SARS-CoV-2/WK-521	original	4.9	≤0.6	≥4.3
SARS-CoV-2/KH-1/2021	original	5.0	≤0.6	≥4.4
hCoV-19/Japan/QK002/2020	alpha	4.2	≤0.6	≥3.6
hCoV-19/Japan/TY8-612/2021	beta	4.8	≤0.6	≥4.2
hCoV-19/Japan/TY7-501/2021	gamma	4.4	≤0.7	≥3.7
SARS-CoV-2/KH-25/2021	delta	4.9	≤0.6	≥4.3

Aliquots (12 μL) of the virus were mixed with 49 volumes of CAC-717 or distilled water and incubated for 5 min. ^1^ The substitutions of viral titers with/without CAC-717 treatment are shown as the log_10_ reduction. Representative data of two independent experiments are shown.

**Table 3:** Effect of pH on virus activity.

pH	Sensitization Time (s)	Viral Titer (TCID_50_/mL)
5	300	4.8 ± 0.2 ^1^
7.5	300	4.9 ± 0.4
10	300	5.0 ± 0.2
11	300	4.4 ± 0.1
12	300	3.6 ± 1.4
12.4	300	≤0.5 ± 0
	60	≤0.5 ± 0
	30	≤0.8 ± 0.6
13	300	≤0.5 ± 0
	60	≤0.5 ± 0
	30	≤0.5 ± 0

Aliquots of virus (SARS-CoV-2/WK-521, 10^7.0 ± 0.3^ TCID_50_/mL) were mixed with nine volumes of pH-adjusted DMEM (from pH 5 to 13) and incubated at 20 °C. ^1^ Mean ± standard deviation of three independent experiments.

**Table 4:** Virucidal effect of CAC-717 with organic matter (BSA).

Disinfectant	BSA	Viral Titer (TCID_50_/mL)	Log_10_ Reduction
CAC-717	+	≤0.5 ± 0 ^1^	4.3 ± 0.1
	−	≤0.5 ± 0.1	4.3 ± 0.1
Control	−	4.8 ± 0.1	−

Aliquots of virus (SARS-CoV-2/WK-521, 10^7.0 ± 0.3^ TCID_50_/mL) were mixed with nine volumes of 5% BSA and incubated at 20 °C for 5 min. The sample was subsequently mixed with CAC-717 or DMEM. ^1^ Mean ± standard deviation of three independent experiments.

## Abbreviations

SARS-CoV-2	severe acute respiratory syndrome coronavirus-2
COVID-19	coronavirus disease 2019
DMEM FBS	Dulbecco’s modified Eagle’s medium fetal bovine serum
BSA TCID_50_,	bovine serum albumin tissue culture infectious dose
HEPES	4-(2-hydroxyethyl)-1-piperazineethanesulfonic acid
